# The “Propofol Battery”: A Teaching Framework for Understanding Multi-compartment Pharmacokinetics

**DOI:** 10.7759/cureus.112130

**Published:** 2026-07-06

**Authors:** John O'Shea, Yulia Gamper

**Affiliations:** 1 Anesthesiology, College of Anesthesiologists of Ireland, Dublin, IRL

**Keywords:** context-sensitive, infusion, pharmacokinetics, pharmacology, propofol, remifentanil

## Abstract

Understanding propofol pharmacokinetics is fundamental to anesthesiology practice, yet traditional multi-compartment models remain challenging for many learners to apply in clinical settings. While these models underpin pharmacological precision and target-controlled infusion systems, they often fail to translate into intuitive bedside reasoning for trainees and non-anesthesiologists. This disconnect contributes to difficulties in explaining common clinical observations, such as the differing recovery profiles following a bolus dose versus a prolonged infusion.

We propose the “propofol battery” as a complementary teaching framework designed to bridge this gap. By conceptualizing drug distribution and accumulation as processes of “charging” and “discharging,” this heuristic simplifies complex pharmacokinetic behavior into an accessible mental model. A bolus dose is represented as minimal charging, producing a transient clinical effect due to rapid redistribution, whereas prolonged infusion results in progressive “charging” of peripheral compartments, with recovery dependent on the time required for redistribution and elimination as steady state is approached.

The model preserves core pharmacokinetic principles while reducing cognitive load, enabling learners to better predict clinical effects and apply pharmacological reasoning in real time. Contrasting agents such as remifentanil, with rapid metabolism and relative context insensitivity, further illustrate the framework's utility.

The “propofol battery” offers a practical, intuitive adjunct to traditional teaching, supporting the development of a functional understanding of propofol pharmacokinetics and improving the translation of pharmacokinetic theory into bedside practice.

## Editorial

The pharmacokinetics of propofol, typically taught through multi-compartment models, remain a foundational yet persistently challenging concept in anesthesiology training [1]. While anesthesiologists need to develop a fundamental understanding of propofol pharmacokinetics, it may not be pragmatic for all healthcare providers who use propofol in clinical practice (e.g., critical care nurses, emergency medicine physicians, intensivists, and procedural specialists) to develop an equally comprehensive understanding of these concepts.

It is our experience that many trainees can recite the model yet struggle to answer practical questions at the bedside: Why does a patient wake quickly after a bolus but slowly after a prolonged infusion? Why does infusion duration matter more than total dose [2]? This disconnect highlights a broader issue in medical education: the gap between formal models and usable mental frameworks [3,4].

To address this, a complementary teaching heuristic may be useful: the “propofol battery.” By reframing drug distribution and accumulation as a process of “charging” and “discharging,” this model offers a cognitively accessible bridge between theory and practice.

The educational problem: when models do not translate

The traditional compartment model is indispensable for target-controlled infusion (TCI) systems and pharmacological precision. However, from an educational perspective, it presents several challenges. It requires simultaneous tracking of multiple compartments with bidirectional drug movement, relies on abstract rate constants that may lack intuitive meaning for those who infrequently use propofol, and separates mathematical understanding from clinical prediction [5].

As a result, trainees and non-anesthesiologists often develop only a superficial familiarity with the model without achieving a functional understanding. They may know what the compartments are but not how to apply the concept [6,7].

The “propofol battery” as a cognitive scaffold

The “propofol battery” reframes multi-compartment pharmacokinetics into a single, intuitive concept: drug accumulation as stored charge. The body acts as a battery system, propofol administration provides the input current, tissues function as storage reservoirs, and recovery represents discharge over time.

This analogy does not discard the underlying pharmacology; rather, it compresses it into a form that is easier to manipulate mentally, particularly for early learners.

Teaching the bolus: minimal charging

According to the traditional multi-compartment model of propofol pharmacokinetics, a bolus induction dose of 1-2 mg/kg [8] initially results in a high plasma (V1) concentration and, consequently, a high effect-site concentration of propofol, producing hypnosis. The drug then undergoes rapid distribution to highly perfused tissues (initial t½ α ≈ 1-2 min) [8], lowering the plasma and effect-site concentrations and leading to prompt clinical awakening [9].

In our proposed model, this can be conceptualized as plugging in a cell phone with an empty battery for 30 seconds. The phone will turn on and may stay on for a few minutes, but will then turn off again because the battery has not been sufficiently charged. The phone being on can be thought of as the clinical hypnotic effect of propofol on the patient.

Teaching infusions: progressive charging

According to the traditional model, a prolonged infusion of propofol results in the redistribution of significant amounts of drug into the muscle and fat compartments (V2 and V3) [10]. This drug must then undergo elimination, with a t½ γ of approximately 71 min [11]. Consequently, a prolonged infusion of propofol results in a longer context-sensitive half-time [2,10].

In our proposed model, this can be thought of as leaving a cell phone with an empty battery plugged into its charger for several hours. Not only will the phone turn on, but it will also stay on for longer because its battery has been charged.

Likewise, a patient receiving a prolonged infusion of propofol can be thought of as having their propofol battery charged. Just as the phone does not turn off immediately when unplugged, the patient will not wake up rapidly, and the clinical effects of propofol will persist for a duration that depends on the length of time the infusion was administered, similar to the rate of battery discharge after the phone is unplugged.

Much like a cell phone battery, the conceptual propofol battery has a practical upper limit to its charge. Over time, drug accumulation approaches a steady state as distribution into the peripheral compartments and elimination reach equilibrium. Beyond this point, continued infusion does not substantially prolong wake-up time.

Remifentanil: what’s different?

Unlike propofol, remifentanil is rapidly hydrolyzed by plasma and non-specific tissue esterases to an inactive metabolite [12]. It is therefore considered relatively "context insensitive," meaning that the offset of its clinical effect is expected to be equally rapid regardless of the duration of infusion [12].

Mapped to our model, the human body can be considered to contain no remifentanil battery (or only a very small one). We can use the analogy of a desktop computer or television, which does not utilize a battery. The device turns on when plugged in but rapidly turns off when unplugged, regardless of how long it has been plugged in.

Conclusion

This model may reduce cognitive load, enhance clinical transfer, and support layered learning from intuition to precision.

The “propofol battery” offers a complementary approach that translates complex pharmacokinetics into an intuitive, clinically useful framework, helping to bridge the gap between theory and bedside understanding.
